# HIV prevalence and incidence in a cohort of South African men and transgender women who have sex with men: the Sibanye Methods for Prevention Packages Programme (MP3) project

**DOI:** 10.1002/jia2.25591

**Published:** 2020-10-01

**Authors:** Patrick S Sullivan, Nancy Phaswana‐Mafuya, Stefan D Baral, Rachel Valencia, Ryan Zahn, Karen Dominguez, Clarence S Yah, Jeb Jones, Lesego B Kgatitswe, AD McNaghten, Aaron J Siegler, Travis H Sanchez, Linda‐Gail Bekker

**Affiliations:** ^1^ Rollins School of Public Health Emory University Atlanta GA USA; ^2^ DVC Research and Innovation Office North West University Potchefstroom South Africa; ^3^ Bloomberg School of Public Health Johns Hopkins University Baltimore MD USA; ^4^ Wits Reproductive Health and HIV Institute Faculty of Health Sciences University of the Witwatersrand Johannesburg South Africa; ^5^ School of Health Systems and Public Health University of Pretoria Pretoria South Africa; ^6^ Human Sciences Research Council of South Africa Port Elizabeth South Africa; ^7^ Desmond Tutu HIV Center University of Cape Town Cape Town South Africa

**Keywords:** HIV, men who have sex with men, sexually transmitted infections, pre‐exposure prophylaxis, HIV prevention, cohort studies

## Abstract

**Introduction:**

Men who have sex with men (MSM) and transgender women (TGW) are at increased risk for acquiring HIV, but there are limited HIV incidence data for these key populations in Africa. Understanding HIV prevalence and incidence provides important context for designing HIV prevention strategies, including pre‐exposure prophylaxis (PrEP) programmes. We describe HIV prevalence, awareness of HIV infection, HIV incidence and associated factors for a cohort of MSM and TGW in Cape Town and Port Elizabeth, South Africa.

**Methods:**

From 2015 to 2016, MSM and TGW in Cape Town and Port Elizabeth were enrolled and prospectively followed for 12 months, receiving a comprehensive package of HIV prevention services. HIV testing was conducted at baseline and at follow‐up visits (targeted for three, six and twelve months). All HIV‐negative PrEP‐eligible participants were offered PrEP enrolment during the first four months of study participation. We determined HIV prevalence among participants at baseline, and incidence by repeat screening of initially HIV‐negative participants with HIV tests at three, six and twelve months.

**Results:**

Among 292 participants enrolled, HIV prevalence was high (43%; 95% CI: 38 to 49) and awareness of HIV status was low (50%). The 167 HIV‐negative participants who were followed prospectively for 144.7 person‐years; nine incident HIV infections were documented. Overall annual incidence was 6.2% (CI: 2.8 to 11.8) and did not differ by city. Annual HIV incidence was significantly higher for younger (18 to 19 years) MSM and TGW (MSM: 21.8% (CI: 1.2 to 100); TGW: 31.0 (CI: 3.7, 111.2)). About half of participants started PrEP during the study; the annual incidence of HIV among 82 (49%) PrEP starters was 3.6% (CI: 0.4, 13.1) and among those who did not start PrEP was 7.8% (CI: 3.1, 16.1).

**Conclusions:**

HIV incidence was high among MSM and TGW in the context of receiving a comprehensive package of prevention interventions and offering of PrEP. PrEP uptake was high; the observed incidence of HIV in those who started PrEP was about half the incidence of HIV in those who did not. Future implementation‐oriented studies should focus on decisions to start and continue PrEP for those at highest risk, including young MSM.

## 1 INTRODUCTION

HIV disproportionally impacts men who have sex with men (MSM) globally, but data about the impact of HIV in MSM [1] and transgender women (TGW) [2] in sub‐Saharan Africa are limited. Most data about the HIV burden among MSM are prevalence data [1]. Understanding HIV epidemics among MSM and TGW in sub‐Saharan Africa requires additional data about HIV burden, inclusive of prevalence and incidence data and appropriately stratified to illustrate heterogeneity within key populations.

Among MSM across sub‐Saharan Africa, there has been observed heterogeneity in risk and needs for prevention services [3]. Most available HIV incidence estimates emerged from biomedical efficacy trials and a few dedicated cohorts of MSM [4, 5]. HIV incidence estimates have been consistently high, from 6% to 20% in control arms and the cohort studies [4, 6, 7, 8]. Moreover, these data have been collected in settings where research capacity is higher and intervention trials have been conducted [9] and from cities where there are more developed gay communities and gay‐friendly HIV prevention and care resources. We need to understand how HIV epidemics in areas with more gay‐friendly services differ from and are similar to epidemics in places where healthcare services are usually delivered through government health clinics.

Outside sub‐Saharan Africa, data have consistently demonstrated higher HIV incidence among younger MSM [10]. To date, there are limited studies describing younger MSM given the complex ethical and legal barriers across sub‐Saharan Africa. In most sub‐Saharan African countries homosexuality is illegal [11], frustrating access to appropriate care [12]. Homosexuality is not criminalized in South Africa, but stigma is widespread, including in healthcare settings, and can discourage disclosure of sexual orientation in healthcare settings [13]. Characterizing appropriate HIV intervention strategies requires studying the differences in HIV risks across the lifespan for MSM by evaluating age‐specific HIV prevalence and incidence.

To contribute data about age‐diverse MSM, TGW and MSM living in different urban settings, we analysed data from the Sibanye Health Project [14, 15], a pilot study of a combination package programme of behavioural, biomedical and community‐led HIV interventions among MSM in Cape Town and Port Elizabeth, South Africa from 2015 to 2016. For the study piloting of provision of combination HIV prevention services, we screened MSM and TGW in Cape Town and Port Elizabeth for HIV status, and prospectively followed a cohort of 167 HIV‐negative MSM and TGW for 12 months.

## 2 METHODS

### 2.1 Study design and population

Data are from the Sibanye Health Project, a pilot study of the acceptability and uptake of a comprehensive combination package of HIV prevention services from 2015 to 2016. The process of developing the package of services has been previously described [14]. The final package included condoms with condom‐compatible lubricant choices, HIV testing with risk‐reduction counselling, couples HIV testing and counselling, screening for sexually transmitted infections (STIs), pre‐exposure prophylaxis (PrEP) for interested and eligible participants, and non‐occupational post‐exposure prophylaxis (PEP) for those with an exposure at high risk for transmission of HIV.

For the pilot study, MSM and TGW in Cape Town and Port Elizabeth, South Africa were recruited from February to September 2015 through community outreach, MSM social gatherings and events, MSM dialogues at homes of peers, social media advertisements, contact of participants in previous prevention studies, walk in, utilization of MSM friendly organizations/clinics, networking and education about the project with major community stakeholders and referral by peers. All participants provided a written informed consent and agreed to participate in the research study and to have their samples used for research purposes.

Eligible participants self‐reported being aged ≥18 years; being assigned male sex at birth; having anal intercourse with a man in the previous 12 months; being residents of the study city; planning to stay in the city for the next year; being able to answer survey questions in English, Xhosa or Afrikaans; being willing to provide contact information; and having a phone line.

The prospective cohort was designed to follow 80% HIV‐negative MSM and 20% MSM living with HIV, but all participants presenting for screening were provided informed consent and completed a baseline study visit consisting of a self‐administered baseline questionnaire, HIV prevention counselling and testing and a clinical assessment. The clinical exam assessed STI history, circumcision status and STI and liver disease symptoms. Laboratory testing for syphilis by RPR testing and urethral and rectal chlamydia and gonorrhoea by PCR testing was offered to all participants.

All prospectively‐enrolled participants were invited and encouraged to attend follow‐up visits scheduled at 3‐, 6‐ and 12‐month post‐baseline, which included surveys, HIV prevention counselling, HIV testing for participants who tested HIV‐negative at their last study visit, a clinical exam assessing STI symptoms and blood and urine collection. Repeat testing for syphilis and urethral and rectal chlamydia and gonorrhoea were offered at 6‐ and 12‐month visits. Participants who completed a study visit within six weeks of the target date were considered to have attended that visit. Eight participants returned >6 weeks after their target 12‐month visit date. These participants are included in the primary incidence analysis.

All participants interested in PrEP were assessed for PrEP eligibility according to then‐current South African national guidelines [16]. Only daily oral PrEP with TDF/FTC was offered during the study. Participants interested in PrEP completed additional screening at either baseline or three months and returned one month later for PrEP initiation (month 1 or 4). Participants on PrEP returned for monitoring visits one month after PrEP initiation (month 2 or 5) and at month 9 to assess creatinine level, liver enzymes, phosphorus, proteinuria, glycosuria, HIV status, medication adherence and to monitor side effects.

At baseline, and follow‐up visits, participants completed a questionnaire with domains including demographics, HIV‐related behaviours and knowledge, sexual history, condom and lubricant use, use of health care services, outness to providers, alcohol and substance use, stigma and the participant’s sexual network. Retention in the cohort was 88% at 12 months when follow‐up ended in September 2016. This study was approved by the IRBs of Emory University and University of Cape Town and the Research Ethics Committee of the Human Sciences Research Council.

### 2.2 Measures

There were two main outcomes: prevalent and incident HIV infection. HIV testing was conducted on whole blood for antibodies to HIV with rapid HIV tests using a serial testing algorithm per South African guidelines [17]. In Cape Town, initial screening was done with Determine™ Rapid HIV‐1/2 test kits (Alere Medical Co. Ltd, Matsudo‐Shi Chiba, Japan). Preliminary positive results were confirmed using a Uni‐Gold™ HIV test (Trinity Biotech PLC., Bray, Ireland). In Port Elizabeth, initial screening was done using Advanced Quality™ Rapid HIV‐1/2 test (InTec Products Inc., Xiamen, China) and confirmed with ABON™ HIV 1/2/O Triline Rapid test (Abon Biopharm, Hangzhou, R.R China). Participants who were HIV negative were followed prospectively for 12 months (or more if the nominal 12‐month visit was delayed), with retesting for HIV at regular follow‐up, PrEP initiation and PrEP monitoring visits [14].

HIV prevalence was the proportion of all MSM screened for HIV at enrolment who had a confirmed positive antibody test. Among MSM living with HIV, awareness was defined as reporting themselves positive in the baseline survey, self‐reporting HIV‐negative to staff who recorded this report on a CRF, and not having a suppressed HIV viral load. Participants who had positive HIV confirmatory testing and undetectable HIV viral load (<20 copies/mL by RT‐PCR analysis) at baseline were considered to be aware of their HIV status.

Participants who seroconverted during the study had HIV viral load and CD4^+^ T‐lymphocyte testing and were referred to a local clinic for antiretroviral treatment initiation. These persons continued to be followed through the end of the study to assess other prevention outcomes (e.g. behaviours and condom use).

Independent variables included age, race, gender identity (male vs transgender or other; transgender was defined as participants who reported male sex at birth and current gender identity as female or transgender), sexual orientation, education level, work or student status, income and circumcision status. The behavioural independent variables included self‐reported receptive condomless anal intercourse with a man in the last three months, number of male partners in the past 12 months, having a female partner in the past 12 months, use of recreational drugs (e.g. marijuana) in the past six months and binge drinking.

### 2.3 Analyses

HIV prevalence and awareness were calculated overall and for subgroups of participants by demographics, behaviours and rectal STI prevalence; 95% confidence intervals for prevalence were calculated using the Wilson procedure without continuity correction [18]. Trends in HIV prevalence and awareness of HIV infection by age were described using a Cochran‐Armitage test for trend. Bivariate prevalence ratios were calculated for factors associated with prevalent HIV infection. An adjusted multivariable logistic regression model included covariates significant at *p* < 0.20 in bivariate analysis. Remaining covariates were considered for collinearity and reported as adjusted prevalence ratios (APRs) with associated 95% CIs using a conditional predicted margins approach [19]. For covariates with *p* < 0.20 in the prevalence model, site‐specific bivariate prevalence ratios were calculated and site‐specific multivariable logistic regression models including these covariates were run.

HIV incidence density was calculated overall and among subgroups using new HIV diagnoses as the numerator and person‐time from baseline to the last completed study visit, other date of loss to follow‐up, or mid‐point between last negative and first‐positive test as the denominator. Participants were allowed to attend follow‐up visits outside the exact date of the three‐monthly intervals. For those who dropped out of follow‐up, we censored their follow time at the time of the last attended study visit. Confidence intervals (alpha = 0.05) for HIV incidence density were calculated using Fisher’s exact test. A Cox proportional hazards model was used to identify factors associated with higher hazard of HIV. Age group, sexual orientation and gender identity were considered as possible predictors of HIV risk. Site and characteristics determined to be associated with HIV incidence via log‐rank tests at alpha = 0.20 were included in the model. Direct age‐standardized estimates of HIV incidence and age‐standardized incidence rate ratios were calculated for Cape‐Town and Port Elizabeth using PROC STDRATE (SAS 9.4, Cary, NC, USA). PrEP was included as a time‐varying exposure. City‐specific HIV incidence density was calculated for variables that were significantly associated with incidence in the Cox proportional hazard model and for PrEP. Timelines for all participants with incident HIV infections were also constructed to identify timing of important prevention (PrEP start and stop) and risk events (new rectal STI diagnoses) relative to time of HIV diagnosis.

## 3 RESULTS

A total of 292 MSM and TGW were enrolled, consented and tested for HIV: 115 were enrolled in Cape Town, and 177 were enrolled in Port Elizabeth. The median age of participants was 24 years (range, 18 to 69); a higher proportion of MSM were young (<20 years) in Cape Town (22%) than in Port Elizabeth (10%). Most were Black African (87%). About half had at least a high school education; most (90%) identified as male and 22 (8%) identified as transgender or other non‐male identified. Enrolment (and assessment of HIV prevalence) occurred from 2015 to 2016; follow‐up (for incidence assessment) occurred from 2015 to 2017.

### 3.1 HIV prevalence

**Figure 1:**
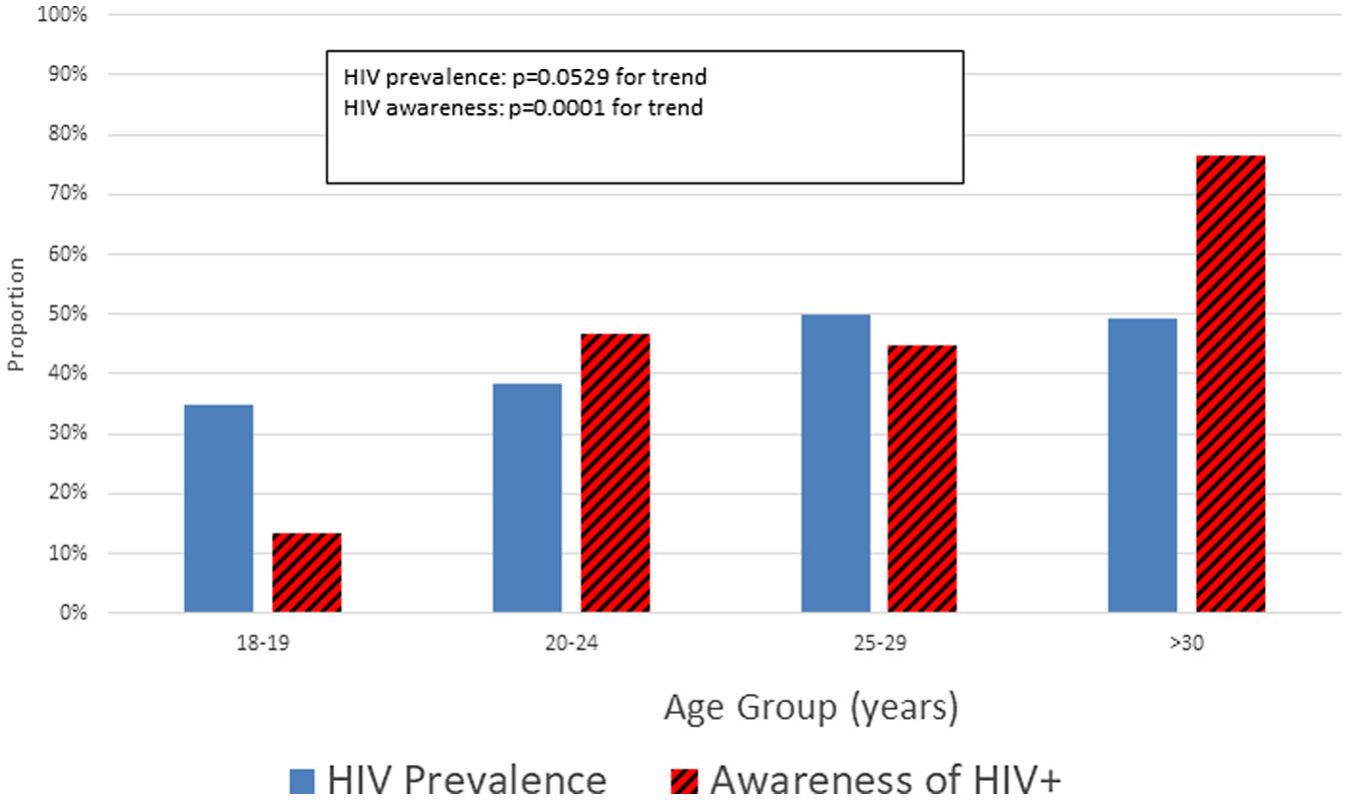
HIV prevalence and awareness of HIV positivity by age among 237 MSM and transgender women, Cape Town and Port Elizabeth, South Africa, 2015 to 2016. MSM, men who have sex with men.

HIV prevalence at enrolment was 43% overall; and was significantly higher (PR = 1.7; 95% CI = 1.2, 2.3; Table 1) in Port Elizabeth (51%) than in Cape Town (30%; Table 1). Self‐reported awareness of HIV‐positive status prior to the study‐delivered HIV testing was 50% overall and was higher in Port Elizabeth(57%; 51/90) than in Cape Town (34%; 12/35). There were significant differences in HIV prevalence by participant demographic characteristics (Table 1), including age (Figure 1).

**Table 1:** HIV prevalence among enrolled South African MSM and transgender women, Sibanye Health Project, 2015 to 2017

	Prevalence Number/total	Prevalence proportion	PR (95% CI)	APR (95% CI) (N = 236)
Total	125/292	43%	–	–
Site				
Cape Town	35/115	30%	Ref	Ref
Port Elizabeth	90/177	51%	1.7 (1.2, 2.3)	2.3 (1.4, 3.7)
Age ranges				
18 to 19	15/43	35%	0.7 (0.5, 1.1)	0.6 (0.4, 1.1)
20 to 24	47/122	39%	0.8 (0.6, 1.0)	0.7 (0.6, 1.0)
25+	63/127	50%	Ref	Ref
Race				
Black	117/254	46%	Ref	Ref
Coloured and other	8/38	21%	0.5 (0.2, 0.9)	0.5 (0.2, 0.9)
Gender identity^a^				
Male	110/263	42%	Ref	Ref
Transgender or other non‐male identified^b^	13/22	59%	1.4 (1.0, 2.1)	1.3 (0.9, 1.8)
Sexual orientation^a^				
Homosexual or gay	105/192	55%	Ref	Ref
Bisexual, straight, other	19/95	20%	0.4 (0.2, 0.6)	0.6 (0.3, 0.9)
Education^a^				
Did not matric/less than high school	54/137	39%	Ref	–
Matric/high school graduate	46/104	44%	1.1 (0.8, 1.5)	
Technical or university education	23/47	49%	1.2 (0.9, 1.8)	
Work or student status^a^				
Full‐time student or full‐time job	50/107	47%	Ref	–
Part‐time student or part‐time job	16/43	37%	0.8 (0.5, 1.2)	
Not a student and no job	58/137	42%	0.9 (0.7, 1.2)	
Income^a^				
No income	61/141	43%	Ref	–
R1 to R4,800	26/64	41%	0.9 (0.7, 1.3)	
R4,801 to R9,600	10/27	37%	0.9 (0.5, 1.5)	
R9,601 to R19,200	6/12	50%	1.2 (0.6, 2.1)	
R19,201 or more	12/27	44%	1.0 (0.7, 1.6)	
Circumcision status				
Full	44/89	49%	1.3 (0.9, 1.8)	0.7 (0.5, 1.1)
Partial	12/36	33%	0.9 (0.5, 1.5)	0.9 (0.6, 1.4)
Uncircumcised	29/74	39%	Ref	Ref
No exam	40/93	43%	1.1 (0.8, 1.6)	0.7 (0.3, 1.2)
Self‐reported receptive condomless anal intercourse in last three months^a^				
Yes	59/93	63%	1.9 (1.4, 2.4)	1.3 (1.0, 1.7)
No	56/165	34%	Ref	Ref
Number of male partners in past 12 months^a^				
0 to 2	56/167	34%	Ref	Ref
3+	69/123	56%	1.7 (1.3, 2.2)	1.4 (1.0, 1.8)
Any female partner in past 12 months^a^				
Yes	12/60	20%	0.4 (0.2, 0.7)	0.8 (0.5, 1.3)
No	113/230	49%	Ref	Ref
Transactional sex in past 12 months^a^				
Yes	16/43	37%	0.8 (0.6, 1.3)	–
No	101/225	45%	Ref	
Any recreational drug use in past six months^a^				
Yes	22/79	28%	0.6 (0.4, 0.9)	0.8 (0.6, 1.1)
No	102/211	48%	Ref	Ref
Binge drinking (5 + drinks) on 5 or more days in past 30 days^a^				
Yes	30/57	53%	1.3 (1.0, 1.7)	1.1 (0.8, 1.5)
No	89/215	41%	Ref	Ref
Rectal STI at baseline				
Yes	31/60	52%	1.3 (1.0, 1.8)	1.2 (0.8, 1.7)
No	52/134	39%	Ref	Ref
No assessment	42/98	43%	1.1 (0.8, 1.5)	1.1 (0.6, 2.0)

APR, adjusted prevalence ratio; PR, Prevalence ratio; R, rand; ref, reference category; STI, sexually transmitted infection.

^a^ Excludes participants with missing data; missing 7 from gender identity measure, missing 5 from sexual orientation, missing 4 from education, missing 5 from work or student status, missing 21 from income, missing 34 from self‐reported receptive condomless anal intercourse in last three months, missing 2 from number of male partners in past 12 months, missing 2 from any female partner in past 12 months, missing 24 from transactional sex in past 12 months, missing 2 from any drug use in past six months, missing 20 from binge drinking.

^b^ includes participants who reported current gender identity as female.

In the multivariable model, HIV prevalence was higher in Port Elizabeth than Cape Town. HIV prevalence was significantly lower in participants aged 20 to 24 compared to ≤25, in non‐black participants, and in participants who identified as bisexual, straight or other. HIV prevalence was significantly higher in participants who self‐reported having ≥3 male sex partners in the past 12 months. No other behaviour or rectal STI diagnosis was associated with HIV prevalence.

There were substantial differences in factors that were significantly associated with HIV prevalence in the city‐stratified analyses (Table 2). For Cape Town participants, there were no significant differences in HIV prevalence by any of the independent measures. For Port Elizabeth participants, there were significant differences in HIV prevalence for participants like what was observed in the overall sample. The exception was that participants who self‐reported receptive condomless anal intercourse in the prior three months were more likely to have prevalent HIV infection compared to those who did not report condomless anal intercourse.

**Table 2:** HIV prevalence among enrolled south African MSM and transgender women, by city, Sibanye Health Project, 2015 to 2016

	Cape Town				Port Elizabeth			
	Prevalence number/total	Prevalence Proportion	PR (95% CI)	APR (95% CI) (N = 104)	Prevalence number/total	Prevalence proportion	PR (95% CI)	APR (95% CI) (N = 151)
Total	35/115	30%			90/177	51%		
Age								
18 to 19	6/25	24%	0.9 (0.4, 2.2)	0.7 (0.3, 1.8)	9/18	50%	0.8 (0.5, 1.3)	0.6 (0.4, 1.0)
20 to 24	16/40	40%	1.5 (0.8, 2.8)	1.4 (0.7, 2.8)	31/82	38%	0.6 (0.4, 0.8)	0.7 (0.5, 0.9)
25+	13/50	26%	Ref	Ref	50/77	65%	Ref	
Race								
Black	30/89	34%	Ref	Ref	87/165	53%	Ref	Ref
Other	5/26	19%	0.6 (0.2, 1.3)	0.5 (0.2, 1.3)	3/12	25%	0.5 (0.2, 1.3)	0.6 (0.3, 1.1)
Sexual orientation								
Gay/homosexual	27/80	34%	Ref	Ref	78/112	70%	Ref	Ref
Bisexual and other	7/32	22%	0.7 (0.3, 1.4)	0.7 (0.3, 1.5)	12/63	19%	0.3 (0.2, 0.5)	0.4 (0.3, 0.7)
Missing					0/2	0%		
Circumcision status								
Full	5/20	25%	0.8 (0.3, 1.8)	0.8 (0.3, 1.8)	39/69	57%	0.9 (0.6, 1.3)	0.9 (0.6, 1.3)
Partial	10/32	31%	1.0 (0.5, 1.9)	0.8 (0.3, 1.7)	2/4	50%	0.8 (0.3, 2.2)	0.5 (0.2, 1.4)
Uncircumcised	19/59	32%	Ref	Ref	10/15	67%	Ref	Ref
No exam	1/4	25%	0.8 (0.1, 4.5)	1.1 (0.1, 8.2)	39/89	44%	0.7 (0.4, 1.0)	0.8 (0.5, 1.2)
Reported receptive condomless anal intercourse in past three months								
Yes	15/36	42%	1.6 (0.9, 2.9)	1.2 (0.6, 2.3)	44/57	77%	1.9 (1.5, 2.6)	1.4 (1.0, 1.8)
No	18/70	26%	Ref	Ref	38/95	40%	Ref	Ref
Missing	2/9	22%			8/25	32%		
Number of male partners in past 12 months								
0 to 2	16/60	27%	Ref	Ref	40/107	37%	Ref	Ref
3+	19/53	36%	1.3 (0.8, 2.4)	1.4 (0.7, 2.6)	50/70	71%	1.9 (1.4, 2.6)	1.4 (1.1, 1.9)

APR, adjusted prevalence ratio; MSM, men who have sex with men; PR, prevalence ratio; Ref, reference category.

### 3.2 HIV incidence

All 167 participants who were HIV negative at baseline were followed prospectively and contributed a total of 145 person‐years (PY) of follow‐up. There were nine incident HIV infections (six in Cape Town; three in Port Elizabeth) during follow‐up. The rate of HIV incidence was 5.3/100 PY (95% CI = 2.1, 10.8) among MSM and 31.0/100 PY (95% CI = 3.7, 111.2) among TGW. Log‐rank tests were significant for age group (*p* < 0.004), sexual orientation (*p* < 0.033) and gender identity (*p* < 0.02). These variables were included in the CPH model (Table 3). The rate of HIV incidence was 6.2/100 PY overall, was 8.8% in Cape Town and was 3.9% in Port Elizabeth. More than half (5/9) of incident HIV infections occurred in participants aged 18 or 19. The rate of HIV incidence in 18 to 19 year olds was 21.8/100 PY, >18 times higher (aHR = 18.5; 95% CI = 1.7 to 196.9) than for participants aged 25 and older (1.8/100 PY). Only gender identity (higher in TGW and other) and sexual orientation (higher in gay than other) were associated significant differences in HIV incidence. City‐stratified analyses were conducted because of the differences in age structure in the two cities, and because of the marked difference in availability of other prevention services in the two cities. In the city‐stratified descriptive analyses, rate of HIV infection among those aged 18 or 19 was 25.0/100 PY in Cape Town and 14.5/100 PY in Port Elizabeth (Table S1). Because of the difference in age distribution between cities, age‐standardized HIV incidence was calculated for each city by standardizing to the South African age structure; the age‐standardized incidence ratio (CT/PE) was 0.96 (CI: 0.55 to 1.67). No participant utilized PEP during follow‐up.

**Table 3:** HIV incidence among baseline HIV‐negative South African MSM and transgender women who were prospectively followed, Sibanye Health Project, 2015 to 2017

	Participants followed	HIV incident infections	Susceptible person‐years	HIV Infections per 100 person‐years (95% CI)	Unadjusted hazard ratio (95% CI)	Adjusted hazards ratio (95% CI) (N = 153)
Total	167	9	144.7	6.2 (2.8, 11.8)	–	–
City						
Cape Town	80	6	68.5	8.8 (3.2, 19.1)	2.1 (0.5, 8.4)	1.4 (0.2, 8.2)
Port Elizabeth	87	3	76.2	3.9 (0.8, 11.5)	Ref	Ref
Age						
18 to 19	28	5	22.9	21.8 (7.1, 51.0)	12.6 (1.5, 108.5)	18.5 (1.7, 196.9)
20 to 24	75	3	65.8	4.6 (0.9, 13.3)	2.6 (0.3, 24.7)	3.2 (0.2, 41.9)
25+	64	1	56.0	1.8 (0.1, 10.0)	Ref	Ref
Race						
Black	137	8	118.4	6.8 (2.9, 13.3)	Ref	–
Other	30	1	26.3	3.8 (0.1, 21.2)	0.6 (0.1, 4.4)	–
Gender Identity						
Male	153	7	133.3	5.3 (2.1, 10.8)	Ref	Ref
Transgender and Other	9	2	6.5	31.0 (3.7, 111.2)	5.5 (1.1, 26.3)	8.7 (1.3, 57.2)
Sexual orientation						
Gay/homosexual	87	8	75.5	10.6 (4.6, 20.9)	7.0 (0.9, 55.8)	11.4 (1.0, 132.2)
Bisexual, straight, other	76	1	66.2	1.5 (0.0, 8.4)	Ref	Ref
Circumcision status						
Full	45	1	40.1	2.5 (0.1, 13.9)	0.2 (0.0, 1.5)	–
Partial	24	1	24.0	4.2 (0.1, 23.2)	0.3 (0.0, 2.4)	–
Uncircumcised	45	5	36.0	13.9 (4.5, 32.4)	Ref	–
No exam	53	2	44.7	4.5 (0.5, 16.2)	0.3 (0.1, 1.8)	–
Education						
Did not matric/less than high school	83	9	69.6	12.9 (6.0, 24.6)	–	–
Matric/high school	58	0	50.9	0.0	–	–
Technical or university education	24	0	22.2	0.0	–	–
Combined work/student						
Full‐time student or full‐time job	57	4	49.1	8.1 (2.2, 20.9)	Ref	–
Part‐time student or part‐time job	27	0	24.2	0.0	–	–
Not a student and no job	79	5	67.5	7.4 (2.4, 17.3)	0.9 (0.2, 3.4)	–
Income						
No income	80	5	66.6	7.5 (2.4, 17.5)	Ref	–
R1 to R4,800	38	1	32.8	3.1 (0.1, 17.0)	0.4 (0.0, 3.6)	–
R4,801 to R9,600	17	1	15.6	6.4 (0.2, 35.7)	0.9 (0.1, 7.5)	–
R9,601 to R19,200	6	0	5.6	0.0 (–)	–	–
R19,201 or more	15	0	14.4	0.0 (–)	–	–
Missing	11	2	9.8	20.3 (2.5, 73.7)	2.7 (0.5, 13.9)	–
On PrEP						
Yes	82	2	55.3	3.6 (0.4, 13.1)	0.7 (0.2, 3.0)	0.6 (0.1, 2.6)
No	85	7	89.4	7.8 (3.1, 16.1)	Ref	Ref

MSM, men who have sex with men; TGW, transgender women; PrEP, pre‐exposure prophylaxis; STI, sexually transmitted infections. Timeline depiction of key events in the study participationof nine MSM and TGW who seroconverted for HIV during studyfollow‐up. Participants were enrolled for a period of 12 months with visits and HIV and STI screening at 3, 6, 9 and 12 months, although the actual dates of study visits varied. Events depicted on the timeline show PrEP‐relatedevents, participant reported sexual risks, STI diagnoses and HIV diagnoses.

**Figure 2:**
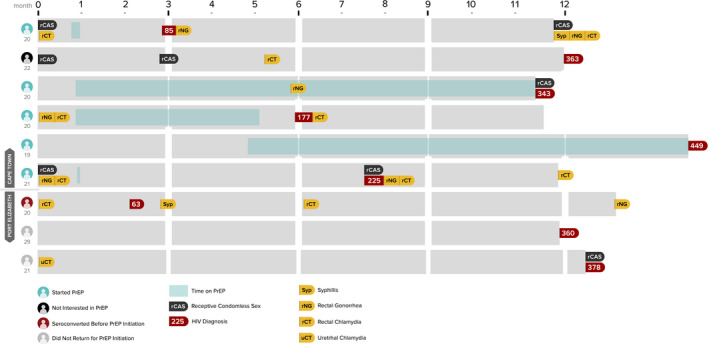
Participant timelines for nine participants who seroconverted for HIV during follow‐up, Cape Town and Port Elizabeth, 2015 to 2017.

The study timelines and relevant clinical events for the nine participants who seroconverted during the study are shown in Figure 2. Only five of the nine used PrEP during the study period; of those, two used PrEP for less than a week (due to side effects), and three reported using PrEP for longer periods, albeit reporting limited adherence. Of the four seroconverting participants who did not use PrEP, one was not interested in PrEP and two were interested in PrEP but did not return for a follow‐up PrEP initiation visit. All but two of the seroconverters had one or more rectal STI diagnoses during the follow‐up period. Most HIV diagnoses were made at the final (12‐month) study visit. Overall, 57% to 72% of participants on PrEP reported adherence across PrEP study visits.

## 4 DISCUSSION

HIV incidence in MSM from two South African cities are consistent with incidence data from elsewhere in Africa (for example, 7% in Kenya; 12.5% in South Africa) [5, 7]. In other settings, comprehensive prevention programmes including PrEP have resulted in lowered risk of infection for MSM [20, 21]. The Sibanye study included the delivery of culturally competent and tailored HIV prevention services for MSM; thus, participants in this study are likely at lower risk for HIV infection than other MSM. Our data reinforce the importance of PrEP to complement existing HIV prevention strategies. They also provide a window to expected challenges in PrEP implementation for South African MSM and TGW.

The data on HIV prevalence and HIV incidence suggest an intense HIV epidemic among MSM in these cities, calling for sustained and comprehensive HIV prevention programmes, with appropriate prioritization of MSM and TGW. In this study, over one‐third of participants aged 18 to 19 years were already living with HIV, suggesting that many new HIV infections in these men and TGW occur during early adolescence [22]. Alternatively, these might have represented perinatal infections that were not previously diagnosed. Therefore, it is important to reach these men and women with information about HIV and MSM‐ and trans‐friendly prevention, HIV screening and health services during their teenage years. HIV prevalence peaked by age 25 to 29, reinforcing the idea that opportunities for prevention are most critical earlier in the life course. Intervening with primary prevention at younger ages also results in lower lifetime transmission risk, providing good value for prevention investment [23]. Similarly, only about one in seven participants who were <20 years old and living with HIV were aware of their HIV infection, and half of participants living with HIV aged 20 to 29 were unaware of their infections. The major change in the prevalence epidemiology for MSM aged ≥30 years was higher awareness of infection (76%). This suggests that developing and promoting culturally relevant HIV testing programmes for MSM and TGW aged 18 to 29 would be especially important in helping those living with HIV to become aware of their infections and gain the benefits of medical care and treatment [24].

HIV incidence data show clear disparities in risk among the MSM and TGW and should inform prevention programmes. Consistent with the interpretation of age‐specific HIV prevalence data, the HIV incidence among MSM and TGW aged 18 to 19 was over 21% per year – a staggering statistic, but one that is consistent with other data from across sub‐Saharan Africa and beyond that show much higher incidence among younger than in older MSM [4, 6, 7, 8]. The concentration of HIV risk among young MSM identified in South Africa is part of a larger global vulnerability of young MSM [10, 25, 26, 27]. There are unique challenges to providing HIV prevention services to MSM and TGW. Ages of sexual debut range widely [28], and many young men engage in sex with male partners for a period of time before identifying as gay or bisexual or accessing gay‐serving or culturally competent health services [29]. Incidence was also higher for MSM who identified as gay and for TGW (versus participants with male gender identity). These findings are consistent with other data from the United States, Asia [30] and Africa [31]. HIV incidence was lower for MSM who identified as straight; for MSM, straight identity might be confounded with sexual positioning (i.e. participants who identified as straight might have been more likely to have an insertive role for anal sex, which is associated with lower per‐act risk of HIV acquisition) [32].

Our data did not document a significant reduction in HIV incidence associated with PrEP use, despite nearly half of the MSM in the cohort engaging in the PrEP programme at some time during their year of prospective follow time. The point estimate suggested a 40% reduction in HIV incidence, but we had a small absolute number of seroconversions and limited power to detect an association of PrEP with incidence. Interpreting our PrEP findings, it is also important to note that no HIV infections occurred in 37 participants who were prescribed PrEP in Port Elizabeth; all new infections among MSM on PrEP were observed in Cape Town. There are several possible reasons that we failed to find a significant protective effect of PrEP in our data. First, our sample size might have been inadequate. The Sibanye study was developed to understand the uptake of specific prevention services, not to identify the effectiveness of PrEP. The current analysis does not account for PrEP adherence; analyses of additional data on self‐reported and laboratory‐measure PrEP adherence might help provide context to our data on PrEP effectiveness. Finally, because PrEP use was not assigned to participants randomly, our data might be subject to confounding by indication, such that the participants at highest risk were most likely to use PrEP. However, we did not observe any differences in PrEP uptake based on measured risk behaviours including receptive anal intercourse in the past three months, participating in exchange sex, or having multiple partners.

A contextual interpretation of the results of our study requires an understanding that the Sibanye Health Project was primarily designed to show the acceptability and uptake of HIV prevention services for MSM and TGW in these two South African cities [14]. As such, participants were offered a broad range of prevention services, including HIV and STI testing, couples risk reduction counselling, free condoms and condom‐compatible lubricant [33] and PrEP services – all provided in culturally competent service settings. Therefore, the finding of over 6% annual incidence in the context of a comprehensive package of prevention offerings delivered by providers trained in serving MSM and TGW should be interpreted as an underestimate of the true risk in these populations, many of whom do not have access to this broad range of prevention services and providers trained to provide culturally congruent care. For example in other settings, incidence in MSM in Africa has been estimated to be as high as 8.6% [4]. This suggests that the components of a comprehensive prevention package including PrEP are crucial, but that delivering these tools in diverse service settings, implementation and getting to scale are outstanding challenges to achieving population‐level reductions in HIV incidence among MSM and TGW [34].

Our study has important limitations, primarily related to the context of the Sibanye Health Project. Our data were collected in 2015 to 2017; even though our data were collected several years ago, recent publications suggest that the high incidence observed in our study might be ongoing in African MSM [5, 7, 9]. Our convenience sample is not representative of all MSM and TGW in the two study cities. We took steps to minimize selection bias (with respect, for example to knowledge of HIV status) by recruiting all MSM and TGW, regardless of perceived HIV status, to the baseline study visit; all participants who screened HIV negative were invited to participate in the incidence cohort. Our data are subject to social desirability or obsequiousness bias [35]. Exposures to risk or protective behaviours were not assigned; therefore confounding by indication or by other measured or unmeasured cofactors might affect our measures of association. Our estimates of HIV incidence were likely artificially lowered by the provision of free high‐quality prevention services, and the higher frequency of interactions with healthcare providers associated with PrEP use. We had 12% loss to follow‐up; it is possible that these participants dropped out of care because they tested positive for HIV elsewhere. One participant who seroconverted for HIV was tested after the 12‐month follow‐up timepoint; his testing was in line with the IRB protocol, and the follow time for his seroconversion appropriately accounted for in the survival analyses.

The results presented here demonstrate two key concepts: the overall high impact of HIV among MSM and TGW globally and the heterogeneity of risks within these groups. The epidemiology of HIV is similar among MSM and TGW in two diverse South African cities and among MSM and TGW in Asia, Europe and North America. In addition, these data highlight the heterogeneity in HIV risks within and across populations even in the context of a general HIV epidemic in South Africa. The current stated goal of the global HIV response is to end new HIV infections by 2030, but the data presented here highlight high HIV incidence, particularly among youth, which challenges the likelihood of achieving this goal. Specifically, the findings that by the age of 19, over a third of young participants were living with HIV and by age 25 about half of participants were living with HIV suggest the importance of tailored responses to address these unmet needs. Traditionally, MSM and TGW have been considered less relevant to HIV responses in more generalized HIV epidemic settings. However, when studied empirically, the HIV transmission dynamics among MSM and TGW in South Africa are like other settings, suggesting that dedicated services for MSM and TGW are fundamental to a comprehensive HIV response.

## 5 CONCLUSIONS

Services for MSM and TGW should be scaled up urgently, consistent with the goals of the Ethekwini Declaration launched in 2019 calling for a renewed focus on HIV in South Africa with specific attention to those most traditionally marginalized.

## COMPETING INTERESTS

No competing interests are declared.

## AUTHORS’ CONTRIBUTIONS

PSS conceived of the study, obtained funding, wrote the protocol, participated in site development, analysed data and wrote the manuscript. RPM participated in writing the protocol, oversaw the Port Elizabeth site activities, and edited the manuscript. SDB participated in writing the protocol, site development, led provider trainings and edited the manuscript. RV participated in site development and management, analysed data and edited the manuscript. RZ participated in site development and management, analysed data and edited the manuscript. KD participated in site development and management for the Cape Town site and edited the manuscript. CY participated in site development and management for the Port Elizabeth site and edited the manuscript. JJ analysed data and edited the manuscript. LK participated in protocol implementation and edited the manuscript. AM wrote the protocol, provided administrative oversight to the study and edited the manuscript. AJS participated in writing the protocol and edited the manuscript. THS participated in writing the protocol and provided overall management for study sites and data activities, and edited the manuscript. LGB participated in writing the grant and protocol, oversaw the Cape Town site and edited the manuscript.

## ABBREVIATIONS

APR, adjusted prevalence ratio; MSM, men who have sex with men; PrEP, pre‐exposure prophylaxis; STI, sexually transmitted infections; TGW, transgender women.

## Supporting information


**Table S1.** HIV incidence among baseline HIV‐negative South African MSM and transgender women who were prospectively followed, by city, Sibanye Health Project, 2015 to 2017
