# Supplementary material for: HIV prevalence and incidence in a cohort of South African men and transgender women who have sex with men: the Sibanye Methods for Prevention Packages Programme (MP3) project
Source: J Int AIDS Soc. 2020 Oct 1;23(Suppl 6):e25591. doi: 10.1002/jia2.25591 (PMC7527763; doi:10.1002/jia2.25591)
Supplement: Supplementary file 1 — Table S1. HIV incidence among baseline HIV‐negative South African MSM and transgender women who were prospectively followed, by city, Sibanye Health Project, 2015 to 2017 [file JIA2-23-e25591-s001.docx]

| **Supplemental Table 1. HIV Incidence Among Baseline HIV-negative South African MSM and Transgender Women who were Prospectively Followed, by City, Sibanye Health Project, 2015-2017** | | | | | | | | | |
| --- | --- | --- | --- | --- | --- | --- | --- | --- | --- |
|  | **Cape Town** | | | |  | **Port Elizabeth** | | | |
|  | **Participants Followed** | **HIV Incident Infections** | **Susceptible Person-years** | **HIV Infections per 100 Person-Years** |  | **Participants Followed** | **HIV Incident Infections** | **Susceptible Person-years** | **HIV Infections per 100 Person-Years** |
| **Total** | 80 | 6 | 68.5 | 8.8 |  | 87 | 3 | 76.2 | 3.9 |
|  |  |  |  |  |  |  |  |  |  |
| **Age** |  |  |  |  |  |  |  |  |  |
| 18-19 | 19 | 4 | 16.0 | 25.0 |  | 9 | 1 | 6.9 | 14.5 |
| 20-24 | 24 | 2 | 20.4 | 9.8 |  | 51 | 1 | 45.4 | 2.2 |
| 25+ | 37 | 0 | 32.0 | 0.0 |  | 27 | 1 | 23.9 | 4.2 |
|  |  |  |  |  |  |  |  |  |  |
| **Gender Identity** |  |  |  |  |  |  |  |  |  |
| Male | 72 | 4 | 63.0 | 6.4 |  | 81 | 3 | 70.3 | 4.3 |
| Transgender and Other | 6 | 2 | 3.6 | 56.2 |  | 3 | 0 | 2.9 | 0.0 |
|  |  |  |  |  |  |  |  |  |  |
| **Sexual orientation** |  |  |  |  |  |  |  |  |  |
| Gay/homosexual | 53 | 6 | 45.6 | 13.1 |  | 34 | 2 | 29.9 | 6.7 |
| Bisexual or other | 25 | 0 | 20.9 | 0.0 |  | 51 | 1 | 45.3 | 2.2 |
|  |  |  |  |  |  |  |  |  |  |
| **Ever started PrEP** |  |  |  |  |  |  |  |  |  |
| Yes | 45 | 2 | 28.7 | 7.0 |  | 37 | 0 | 26.6 | 0.0 |
| No | 35 | 4 | 39.8 | 10.1 |  | 50 | 3 | 49.6 | 6.0 |

MSM: Men who have sex with men; PrEP: pre-exposure prophylaxis
